# Favourable long-term survival of patients with esophageal cancer treated with extended transhiatal esophagectomy combined with en bloc lymphadenectomy: results from a retrospective observational cohort study

**DOI:** 10.1186/s12893-020-00855-z

**Published:** 2020-09-11

**Authors:** Dino Kröll, Yves Michael Borbély, Bastian Dislich, Tobias Haltmeier, Thomas Malinka, Matthias Biebl, Rupert Langer, Daniel Candinas, Christian Seiler

**Affiliations:** 1 Department of Surgery, Campus Charité Mitte and Campus Virchow-Klinikum, Charité-Universitätsmedizin Berlin, Augustenburger Platz 1, 13353 Berlin, Germany; 2 Department of Visceral Surgery and Medicine, Inselspital Bern, Bern University Hospital and University of Bern, 3010 Bern, Switzerland; 3 Institute of Pathology, Department of Clinical Pathology, Inselspital Bern, Bern University Hospital and University of Bern, 3010 Bern, Switzerland

**Keywords:** Esophageal cancer, Extended transhiatal esophagectomy, Long-term survival, En bloc lymphadenectomy, Short-term outcome

## Abstract

**Background:**

Although considered complex and challenging, esophagectomy remains the best potentially curable treatment option for resectable esophageal and esophagogastric junction (AEG) carcinomas. The optimal surgical approach and technique as well as the extent of lymphadenectomy, particularly regarding quality of life and short- and long-term outcomes, are still a matter of debate. To lower perioperative morbidity, we combined the advantages of a one-cavity approach with extended lymph node dissection (usually achieved by only a two-cavity approach) and developed a modified single-cavity transhiatal approach for esophagectomy.

**Methods:**

The aim of this study was to evaluate the outcome of an extended transhiatal esophageal resection with radical bilateral mediastinal en bloc lymphadenectomy (eTHE). A prospective database of 166 patients with resectable cancers of the esophagus (including adenocarcinomas of the AEG types I and II) were analyzed. Patients were treated between 2001 and 2017 with eTHE at a tertiary care university center. Relevant patient characteristics and outcome parameters were collected and analyzed. The primary endpoint was 5-year overall survival. Secondary outcomes included short-term morbidity, mortality, radicalness of en bloc resection and oncologic efficacy.

**Results:**

The overall survival rates at 1, 3 and 5 years were 84, 70, and 61.0%, respectively. The in-hospital mortality rate after eTHE was 1.2%. Complications with a Clavien-Dindo score of III/IV occurred in 31 cases (18.6%). A total of 25 patients (15.1%) had a major pulmonary complication. The median hospital stay was 17 days (interquartile range (IQR) 12). Most patients (*n* = 144; 86.7%) received neoadjuvant treatment. The median number of lymph nodes resected was 25 (IQR 17). The R0 resection rate was 97%.

**Conclusion:**

In patients with esophageal cancer, eTHE without thoracotomy resulted in excellent long-term survival, an above average number of resected lymph nodes and an acceptable postoperative morbidity and mortality.

## Background

Esophageal cancer is one of the most common malignant tumors of the digestive system and has an unfavorable prognosis [1]. In recent decades, careful patient selection, multimodal treatment concepts, and modified surgical strategies have led to better outcomes for patients with locally advanced cancers of the esophagus and the gastroesophageal junction (GEJ); nevertheless, further treatment improvements are needed.

Surgery remains the best curative treatment option for resectable esophageal cancer [2]; however, the optimal surgical technique is not yet defined and remains the topic of an ongoing debate with regard to surgical approaches and techniques (e.g., open vs. minimally invasive vs. robotic and hybrid surgery) and the extent of lymphadenectomy and its influence on short- and long-term outcomes.

Minimally invasive esophagectomy approaches (MIE) and hybrid resections are increasingly used in oncologic surgery for esophageal carcinoma, showing advantages regarding postoperative convalescence. However, a real benefit regarding oncologic safety and long-term survival is still lacking [3–5].

Two large meta-analyses comparing transhiatal (single-cavity approach) and transthoracic (two-cavity approach) routes have attempted to address the debate regarding the best surgical approach [6, 7]. Transthoracic esophagectomy (Ivor Lewis) is believed to benefit long-term survival. Due to better tumor exposure and control, the radicalness of resection and the extent of lymphadenectomy seem to favor a transthoracic approach (TTE) as opposed to the transhiatal approach (THE), which is more focused on decreasing postoperative morbidity (i.e., fewer respiratory complications) and mortality by preventing formal thoracic access and avoiding one-lung ventilation [8–10]. However, most previous studies were retrospective and did not include neoadjuvant treatment options. Although experienced groups have developed many different techniques and approaches, an improvement in the disease-free period or a significant overall survival benefit of these techniques has not yet been demonstrated.

To combine the advantages of TTE and THE, an extended transhiatal esophageal resection through a one-cavity approach including extended transhiatal en bloc-lymphadenectomy (eTHE) and a cervical esophagogastric anastomosis, but not thoracotomy, was introduced. In this descriptive and exploratory retrospective study, we aimed to evaluate patients who were treated in a tertiary care center regarding the benefits and potential limitations of the eTHE technique.

## Methods

Patients undergoing a planned esophageal resection between December 2001 and May 2017 at the Bern University Hospital and University of Bern, Inselspital were reviewed. All patients were consecutively registered in a prospective database, which was then evaluated retrospectively. Patients 18 to 80 years old with resectable esophageal cancer (cT1–3, N0–4, and M0) of the intrathoracic esophagus or GEJ (Siewert type I and Siewert type I-II) treated with eTHE and a cervical anastomosis were eligible for inclusion. Adenocarcinomas, squamous cell carcinomas, and other carcinomas (e.g., mixed adeno-neuroendocrine carcinoma) were included. Patients with benign diseases and those who underwent partial resections of the distal esophagus (*n* = 125) and patients who underwent TTE (*n* = 9) were excluded. The independent cantonal ethics committee approved this study.

### Operative approach

eTHE was performed with a small upper midline laparotomy. The lower mediastinal and paraesophageal dissection was accomplished through opening the diaphragmatic hiatus approximately 7 cm anteriorly. Lymphadenectomy of the celiac trunk (including its branches) along the pancreas, splenic region and perigastric tissue was performed. Continuing the en bloc lymphadenectomy towards the mediastinum and neck, paraesophageal and mediastinal tissue (including pleura, pericardial and paraaortic lymph and fatty tissues) and lymph nodes were removed carefully, avoiding lesions of the thoracic duct via a “transmediastinal shift technique”. To achieve this, a long retractor was used to alternatively elevate/rotate the right and left lungs anteriorly to give free sight and access to the mediastinum and thoracic cavity up to the apex/first rib on both sides (Supp. Figure S2). Rotating the entire mediastinum, anteriorly alternating the lungs on the right and left sides provides an open view and access to all mediastinal structures and paraesophageal tissues, comparable to that achieved with open thoracotomy (Supp. Figure S3) [11]. It was, however, of utmost importance to team up with an experienced anesthesiologist to carefully monitor the blood pressure during this phase of the surgical procedure and to avoid/treat potential iatrogenic hypotension. An additional cardio-respiratory status monitor was placed in the surgeon’s field of view. The aim was to resect an en bloc specimen of the esophagus following the anatomical planes to the mediastinum, comparable to the total mesorectal specimen technique in advanced colorectal surgery. All tissue between the pericardium and the aorta, including the pleura laterally and the tracheal bifurcation, was removed, preferably in one noninjured piece (Supp. Figure S4). The neck was opened through a small left cervical incision, similar to that used for a formal neck dissection, allowing us to perform a lymphadenectomy of levels 3 to 7 while visualizing and sparing the recurrent laryngeal nerve (RLN) and the parathyroid glands [12]. A combined dissection of the remaining short cervical esophagus was performed from the neck and the abdomen. The gastric conduit was dissected via a fundus rotation gastroplasty with successive firings of endoscopic linear staplers on the distal lesser curvature of the stomach [13]. The gastric tube was routed through the hiatus into the neck. Then, a high esophagogastric anastomosis was performed in the neck, preferably with an end-to-side circular stapler (25 mm). A nasogastric tube was placed through the anastomosis under direct vision, and a feeding jejunostomy tube was placed at the end of the procedure. Two-Jackson Pratt drain were placed into both pleura cavities. Finally, the diaphragm was reconstructed.

### Outcomes

A retrospective chart review was performed using a standardized outcome protocol. Patient demographics, details regarding the surgical procedure, use of neoadjuvant chemoradiotherapy (nCRT), tumor-specific variables and survival outcomes were recorded. A routine pathology work-up was performed as recommended [14]. Tumors were classified according to the World Health Orgnaziation classification [15], and staging was performed according to the UICC/American Joint Committee on Cancer (eighth edition) criteria [16].

The primary outcome of this study was 5 year overall survival. Secondary outcomes included perioperative morbidity, reoperations, postoperative all-cause mortality (in-hospital, 30 days, and 90 days) and oncologic efficacy.

The Clavien-Dindo classification was used for the classification of perioperative complications [17]. Postoperative complications included anastomotic leakage (identified clinically or radiographically), respiratory complications (pneumonia or bronchopneumonia confirmed by a computed tomographic scan of the thorax), cardiovascular complications (defined as persistent arrhythmia requiring medical treatment and myocardial infarction), wound infections and other complications (i.e., recurrent nerve injury). Lack of vocal cord function recovery within 6 months after surgery was defined as permanent laryngeal nerve palsy (diagnosed by laryngeal electromyography). Postoperative mortality was defined as death from any cause. All patients were regularly evaluated at the outpatient department. In the first year, follow-up consisted of evaluations every 3 months and every 6 to 12 months in the second, third, fourth, and fifth year postoperatively. Long-term follow-up data were collected by chart review and, in case of missing data, by contacting the general practitioner or the patient directly.

### Statistical analysis

The normality of continuous variable distribution was assessed using histograms, skewness, and the Shapiro–Wilk test. Categorical variables are reported as numbers and percentages, and continuous variables are reported as medians and interquartile ranges (IQRs). Survival was assessed with Kaplan-Meier curves and life table analyses. Analyses were performed using SPSS Statistics version 24 (IBM, Armonk, NY, USA) and STAT Version 16 (StataCorp, College Station, TX, USA).

## Results

### Patients characteristics

Between December 2001 and May 2017, 166 patients were included in the present study. Baseline demographics and clinical characteristics, including age, sex, American Society of Anesthesiologists (ASA) classification, and comorbidities (e.g., heart disease and pulmonary diseases), are displayed in Table 1. Most patients were male (78.3%), and cT3 was the most frequently observed clinical tumor stage (63.9%). A total of 144 patients underwent nCRT (86.7%). Radiation was performed with mainly 45 Gy (*n* = 84) or 50.5 Gy (*n* = 22). Chemotherapy was 5-Fluoruracil and Cisplatin/Carboplatin based with additional Paclitaxel or Doxetaxel. Median follow-up was 29 months (IQR 52).

**Table 1:** Patient characteristics and clinical characteristics

	eTHE (***n*** = 166)
Age (years)	67.0 (13.0)
Gender (male/female)^a^	130/36 (78.3/21.7)
ASA score 2^a^	53 (31.9)
ASA score 3^a^	103 (62.0)
ASA score 4^a^	10 (6.0)
Pulmonary disease^a^	49 (29.5)
Cardiovascular disease^a^	87 (52.4)
Neoadjuvant treatment^a^	144 (86.7)
cT-stage, n (%)	
cT1	14 (8.4)
cT2	44 (26.5)
cT3	106 (63.9)
cT4	2 (1.2)
cN-stage, n (%)	
cNo	38 (22.9)
cN1	97 (58.4)
cN2	30 (18.1)
cN3	1 (0.6)

Values are medians (interquartile ranges) unless indicated otherwise

*eTHE* extended transhiatal esophageal resection with radical bilateral mediastinal en bloc lymphadenectomy, *BMI* body mass index, *ASA* American Society of Anesthesiologists Physical Status Classification System

^a^Values are numbers (percentages)

### Morbidity and mortality

There were no intraoperative deaths. The in-hospital mortality rate was 1.2% (n = 2), and the 30- and 90-day mortality rates were 1.8 and 4.2%, respectively (*n* = 3). The postoperative morbidity is summarized in Table 2. A total of 25 patients (15.1%) had a major pulmonary complication.

**Table 2:** Operative data and postoperative adverse outcomes after eTHE

	eTHE (***n*** = 166)
Operating time (min)	380 (88)
Blood loss (ml)	500 (238)
Clavien-Dindo^a^	
I	32 (19.3)
II	60 (36.1)
III	19 (11.4)
IV	12 (7.2)
V	3 (1.8)
Vocal cord paresis/paralysis^a^	
Transient	18 (10.8)
Permanent	1 (0.6)
Anastomotic leak^a^	19 (11.4)
Reoperation overall^a^	9 (5.4)
Revision for leakage^a^	3 (1.8)
SSI^a^	16 (9.6)
Cardiovascular complications^a^	51 (30.7)
Hospital-acquired pneumonia^a^	21 (12.7)
ARDS^a^	4 (2.4)
In-hospital mortality^a^	2 (1.2)
30-day mortality^a^	3 (1.8)
90-day mortality^a^	7 (4.2)
Total hospital LOS	17 (12.0)

Values are medians (interquartile ranges) unless indicated otherwise

*eTHE* extended transhiatal esophageal resection with radical bilateral mediastinal en bloc lymphadenectomy, *ARDS* acute respiratory distress syndrome, *SSI* surgical site infection, *LOS* length of stay

^a^ Values are numbers (percentages)

### Pathological findings

Adenocarcinoma was the most frequently observed type of tumor (*n* = 114; 68.7%). The remaining cases were squamous cell carcinomas (*n* = 46; 27.7%) and other cancers (*n* = 6; 3.6%). Carcinomas were mainly located in the distal esophagus or the GEJ (82.5%). The median number of resected lymph nodes was 25 (IQR 17). The R0 resection rate with negative margins on final pathologic review was 97% (Table 3). Complete regression of the primary tumor after neoadjuvant therapy corresponding to tumor regression grade Becker 1a was observed in 49 of 144 patients (34%) undergoing multimodal treatment. Eight patients (16.3%) with complete regression of the primary tumor had viable lymph node metastases.

**Table 3:** Histological features after eTHE

	eTHE (***n*** = 166)
Tumor localization, n (%)	
Upper third	1 (0.6)
Middle third	28 (16.9)
Distal third	137 (82.5)
AEG Typ I	101 (73.7)
AEG Typ II	29 (21.2)
Tumor entity, n (%)	
Squamous carcinoma, n (%)	46 (27.7)
Adenocarcinoma, n (%)	114 (68.7)
Others	6 (3.6)
(y) pT-Stage^a^	
ypT0	42 (25.3)
(y) pT1	46 (27.7)
(y)pT2	22 (13.3)
(y)pT3	56 (33.7)
N-Stage^a^	
pN0	111 (66.9)
pN1	32 (19.3)
pN2	16 (9.6)
pN3	7 (4.2)
TNM 8-Stage^a^	
I	89 (53.6)
II	19 (11.4)
IIIA	15 (9.0)
IIIB	30 (18.1)
IVA	7 (4.2)
IVB	6 (3.6)
RO resection	161 (97.0)
No. of resected lymph nodes	25 (17.0)
Complete response (Becker 1a)^a^	49 (34.0)

Values are medians (interquartile ranges) unless indicated otherwise

*eTHE* extended transhiatal esophageal resection with radical bilateral mediastinal en bloc lymphadenectomy

^a^ Values are numbers (percentages)

### Overall survival

**Fig. 1:**
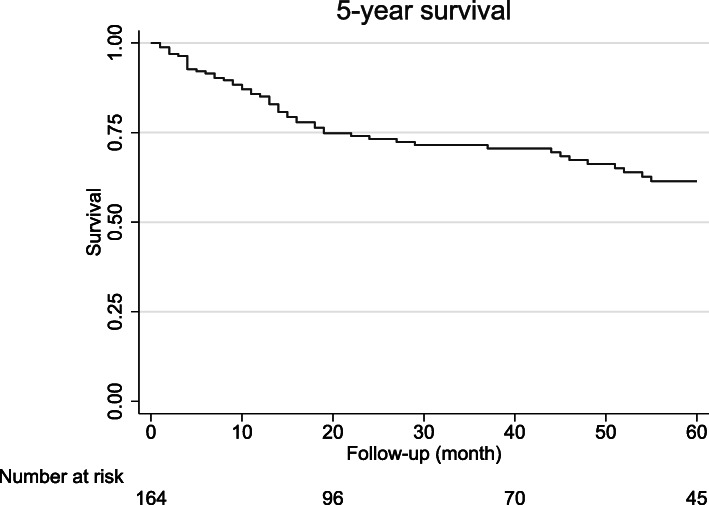
Kaplan-Meier plots of the estimated overall survival for up to 5 years after cancer resection

**Fig. 2:**
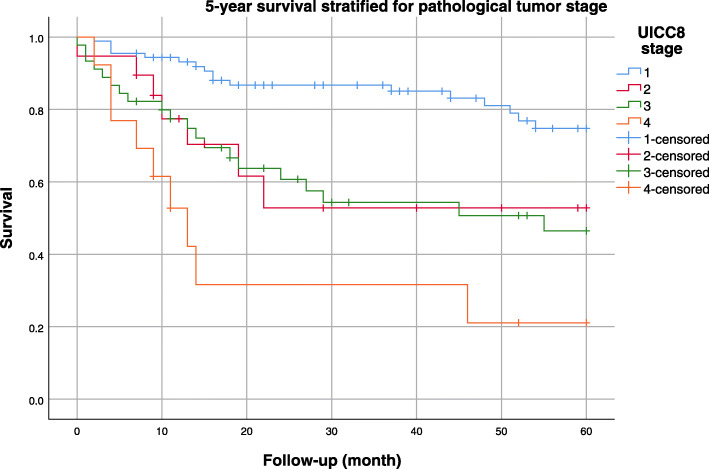
Five-year survival stratified by pathological tumor stage. _UICC, Union for International Cancer Control_

**Fig. 3:**
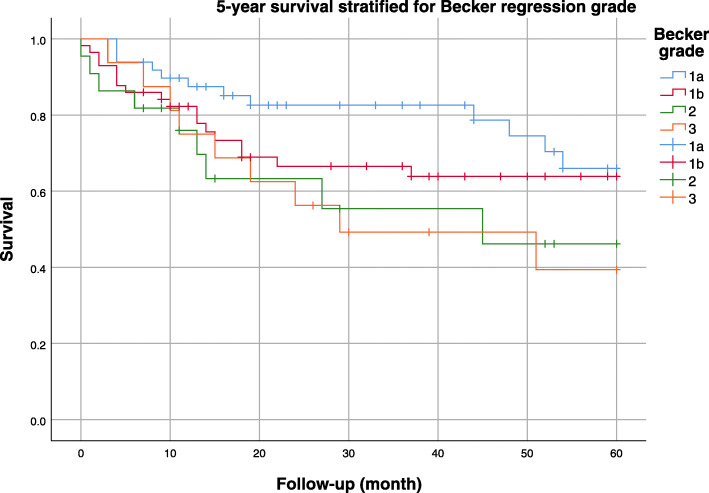
Five-year survival stratified by Becker regression grade

The 1-year survival rate was 84%, the 3-year survival rate was 70%, and the 5-year survival rate was 61%. The overall survival curves taking into account all tumor stages are presented in Fig. 1. Figure 2 shows the Kaplan-Meier survival curves stratified by pathology stage. The best 5-year survival was observed in pathology stage I patients and in patients with a complete response to neoadjuvant treatment (Fig. 3). Supplemental Fig. S1 shows the Kaplan-Meier survival curves stratified by tumor type (adenocarcinoma vs. squamous cell carcinomas).

## Discussion

The optimal approach to esophagectomy, regardless of whether an open or minimally invasive (hybrid/robotic) technique is used, is challenging and controversial. Debates regarding postoperative morbidity and long-term oncological outcomes are ongoing.

This single-institution review of the open eTHE technique for esophageal cancer demonstrates the low perioperative morbidity and mortality of this approach with above-average overall long-term survival.

The transhiatal technique was introduced in the 1970s by Orringer et al., and its efficacy was demonstrated shortly after its introduction [18]. The advantage of the transhiatal technique is the avoidance of a two-cavity approach, omitting thoracotomy. The aim was a) to reduce surgical trauma and minimize pulmonary complications, b) to establish a cervical esophageal anastomosis in a position where a postoperative leak can be treated easily, and c) to avoid a mediastinal anastomosis with the risk of mediastinitis and sepsis.

Anastomotic leaks occur in 9–14% of cases, and some small studies have reported an incidence as low as 1.8% [18, 19]. In our study, the leak rate was 11.4%, with 1.8% of cases requiring surgery and drainage, and the incidence of temporary RLN injury was 10.8%. One patient suffered from permanent RLN (0.6%), which is comparable to the frequency reported in a meta-analysis of more than 5000 patients [6]. The pulmonary complication rate was acceptable (12.4%) and comparable to results from the Esophagectomy Complications Consensus Group with pneumonia rates of 11.4% [20].

The overall postoperative 30-day mortality rate was 1.8%, which was similar to that in other MIE studies [21]. The 90-day mortality rate was 4.2%, which was less than half of the rate reported in the National Cancer Database (8.9%) [22].

The conclusion of a subsequent analysis in the HIVEX trial [23] was that TTE was the preferred technique, whereas THE should be considered only in patients with a carcinoma located at the GEJ or in patients with a poor performance status. This conclusion could not be confirmed in the present study. The baseline characteristics of patients in our cohort (e.g., patient population age, gender, comorbidities, neoadjuvant therapy, tumor histology, site, and stage) are comparable to those of other single-center esophagectomy research groups at major academic medical centers and multi-institution studies of esophagectomy [10, 24, 25]. Although the ASA classification might be a subjective parameter, the number of patients with ASA III and ASA IV was significantly higher in our cohort than in other studies [26].

Most importantly, with eTHE, the factors regarding oncologic efficacy, such as lymph node harvest, complete resection rate (R0 of 97%), and long-term survival outcomes, were no compromised compared to those of other open or laparoscopic approaches. A recent study comparing open TTE to minimally invasive TTE showed no significant difference in long-term survival for up to 3 years (40.2% (±6.9) vs. 35.9% (±6.8%)) [25]. Our long-term outcomes are superior with overall survival rates at 3 and 5 years of 70 and 61%, respectively [9, 25–27]. There are probably various reasons for this discrepancy, which will be discussed in the following section.

The oncologic appropriateness of the THE has been questioned because critics argue that a thoracic mediastinal lympadenectomy cannot be performed adequately with the transhiatal approach. A population-based cohort study in the Netherlands demonstrated a survival advantage was achieved with an increasing number of removed lymph nodes (HR 0.84; 0.78–0.90 per 10 more lymph nodes removed). In the transthoracic resection approach, a median of 19 (range 14–25) lymph nodes was removed, whereas considerably fewer lymph nodes were removed during transhiatal resection (median 12, range 8–17). The median overall number of lymph nodes resected in our study cohort, including patients who underwent nCRT (86.7%), was 25, which is higher than the average number reported [27] and disproves the statement that a transhiatal approach allows lymph node dissection to only a limited extent. Peyre et al. even suggested that the optimal threshold for this survival benefit was the removal of at least 23 nodes, and the surgical procedure to achieve this number with a high likelihood was en bloc resection [28].

By opening the diaphragm anteriorly and thus having full visibility of all essential structures in the mediastinum, the advantages of TTE (radicalness) and THE (low morbidity, one-cavity approach and no need for one-lung ventilation) were combined by the eTHE.

A complete en bloc total mesorectal excision, the emerged standard of care in colorectal surgery, has been defined as the “complete removal of the lymph node-bearing mesorectum along with its intact enveloping fascia” [29]. The same authors have also shown that an incomplete total mesorectal excision is associated with an increased risk of recurrence and a decreased overall survival [30]. Preparation along embryonic layers of the upper gastrointestinal tract is also attracting increasing attention among oncologists, although its scientific basis and superiority have not yet been proven. Following the principles of total mesorectal resection, Cuesta et al. described the “meso-esophagus” for the first time [31]. This principle of respecting the planes surrounding the specimen being transferred to esophageal cancer surgery might be crucial and may have contributed to the excellent long-term results in our eTHE series with en bloc resection and high numbers of resected lymph nodes. The use of long laparoscopic instruments was precious and enabled a save and radical dissection and resection in the mid and upper mediastinum along with the landmarks also for tumors in the middle third of the esophagus. However, there are certain limitations for tumours (bulky tumours, metastatic lymph nodes) in the middle third for the eTHE technique, and a classic open IVOR Lewis approach is also a good option. However, the number of carcinomas in the upper third (*n* = 1, 0.6%) of the esophagus was low in our study. High cervical esophagus carcinoma, non-responding to radiochemotherapy were sporadic and could be treated with total laryngo-esophagectomy, but no patient underwent such a procedure during the period. We usually performed a 2-field lymphadenectomy up to the level of the vena azygos, but in single cases, lymphadenectomy of the supraaortal lymph nodes via deep cervical access is also possible.

The eTHE technique in our study was pursued in order to reduce the pulmonary complication rate of an open transthoracic esophageal resection by avoiding thoracotomy and single lung ventilation. Open transthoracic esophagectomy is burdened by a postoperative pneumonia rate of 12–55% even in recent randomized trials [25, 32, 33]. Besides the surgical trauma of the resection itself, the surgical access trauma seems crucial for the pulmonary function, as all randomized trials comparing open and minimally invasive esophagectomy display a substantial reduction of the pulmonary complication rate in the minimally invasive cohort. Very interestingly, this holds for all types of minimally invasive access, i.e., a combination of intrathoracic of cervical anastomoses like in the TIME trial [25], fully robotic access like in the Dutch ROBOT trial published by van der Sluis et al. [32], and even for the hybrid access as published by French FREGAT group in the MIRO trial [33]. Comparing the results of our study to the outcome of the MIRO trial, short-term results were comparable about rates of major postoperative complications according to Clavien-Dindo classification. In our series, postoperative pneumonia and ARDS rate was 12.7 and 2.4%, respectively, as compared to 12.8 and 7.8%, respectively, in the hybrid-procedure group of the MIRO trial [33]. Both results compare at least similar to the 28% pneumonia rate of the ROBOT trial [32] and the 12% pneumonia rate of the TIME trial [25]. Therefore, the inherent pulmonary complication rate of an esophageal resection seems to be significantly aggravated by the destruction of the primary and auxiliary respiratory musculature at the chest and abdominal level through an open two-cavity Ivor-Lewis resection. Even avoiding one of these resections by using either a minimally invasive approach or – in our case – a transhiatal access, prevents the body from the “double hit” of the open esophagectomy and reduces postoperative pulmonary failure.

Our data, together with the studies mentioned above, seem to indicate that in the setting of esophagectomy, firstly, it seems irrelevant which side of the diaphragm remains without a long incision. Secondly, the surgical trauma and postoperative morbidity of the removal of the esophagus itself seems to transfer a “baseline” morbidity, which overlays the presumed additional benefit of a total minimally invasive procedure, resulting in favorable postoperative complication rates in both our and the MIRO hybrid cohort compared to the Dutch ROBOT and the TIME total minimally invasive patient cohort. In conclusion, the presented modified eTHE technique resulted in limited surgical trauma avoiding thoracotomy with consecutive pulmonary morbidity and without jeopardizing the radicality of the procedure since extensive lymphadenectomy was still feasible.

In the current era of MIE, the advantage of an open eTHE with a small supraumbilical incision may become more significant if long-term oncological outcomes of MIE are similar to those of open techniques. Furthermore, robotic and mediastinoscope assisted-transhiatal esophagectomy has found its place among minimally invasive techniques and is becoming more popular [34, 35]. Mori et al. developed a new robotic technique, “nontransthoracic esophagectomy”, with promising results. The authors described the performance of an improved transhiatal nodal dissection without the disadvantages of a thoracic approach. While this technique potentially represents an interesting alternative to current surgical procedures, it needs to be further evaluated, even for advanced stages of cancer.

Beyond the selection of the surgical technique, the implementation of multimodal therapy, the extent of surgical resection, the experience of the surgeon, and the operative volume as well as improvements in critical care management in the hospital may also play important roles in both short-term and long-term efficacy and outcomes [18, 36, 37].

The limitations of this study are those commonly associated with retrospective studies. There following confounding factors were present: differences in patient characteristics, increasing surgical experience, expertise in postoperative management and critical care and evolution over this long study period. Other limitations of the present study are the relatively small number of patients analyzed and the single-center approach.

## Conclusions

In conclusion, this single-center cohort study demonstrates that extended transhiatal esophageal resection without thoracotomy is associated with low peri- and postoperative morbidity and mortality. With eTHE, radical resection including extensive mediastinal lymphadenectomy is feasible, and this oncologically sound procedure resulted in above-average long-term survival rates compared to those achieved with other techniques described in the literature.

## Supplementary information

**Additional file 1: Supp. Fig. S1.** Five-year survival stratified by tumor type.

**Additional file 2: Supp. Fig. S2**. En bloc resection of the esophagus and periesophageal tissue, including all tissue between the aorta and pericardium (and the pleura, bilaterally).

**Additional file 3: Supp. Fig. S3.** View of pulmonal arteries completely cleared from periesophageal tissue by elevating the pulmonary hilus anteriorly to gain access to the hilar structures, esophagus, and left lung.

**Additional file 4: Supp. Fig. S4.** View of the tracheal bifurcation/aortopulmonal window.

## Data Availability

The datasets generated and analyzed during the current study are not publicly available as the data also forms part of an ongoing study but are available from the corresponding author on reasonable request.

## Abbreviations

AEG: Esophagogastric junction
ASA: American Society of Anesthesiologists
eTHE: Extended Transhiatal esophageal resection with radical bilateral mediastinal en bloc lymphadenectomy
GEJ: Gastroesophageal junction
IQR: Interquartile range
MIE: Minimally invasive esophagectomy approach
nCRT: Neoadjuvant chemoradiotherapy
RLN: Recurrent laryngeal nerve
THE: Transhiatal approach
TTE: Transthoracic approach
UICC: Union internationale contre le cancer
