# Supplementary figures and images for: Favourable long-term survival of patients with esophageal cancer treated with extended transhiatal esophagectomy combined with en bloc lymphadenectomy: results from a retrospective observational cohort study
Source: BMC Surg. 2020 Sep 11;20:197. doi: 10.1186/s12893-020-00855-z (PMC7488573; doi:10.1186/s12893-020-00855-z)

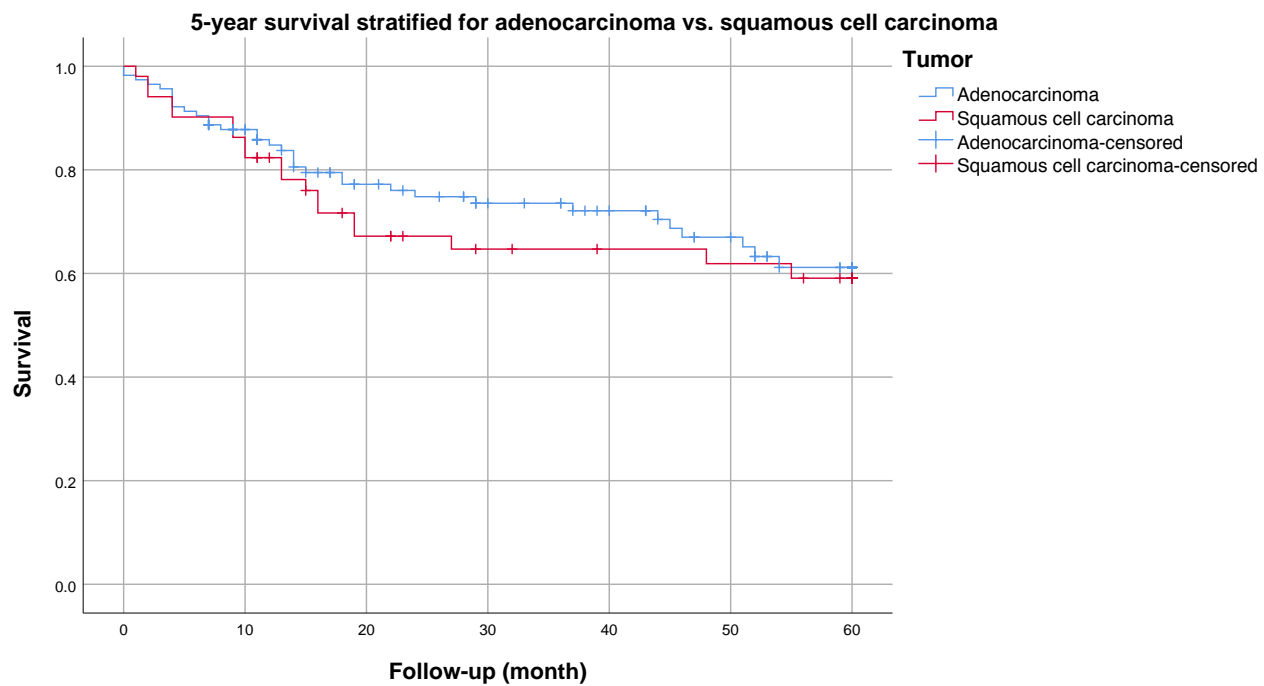

Supplement: Supplementary file 1 — Additional file 1: Supp. Fig. S1. Five-year survival stratified by tumor type. [file 12893_2020_855_MOESM1_ESM.pdf]

***

**

*


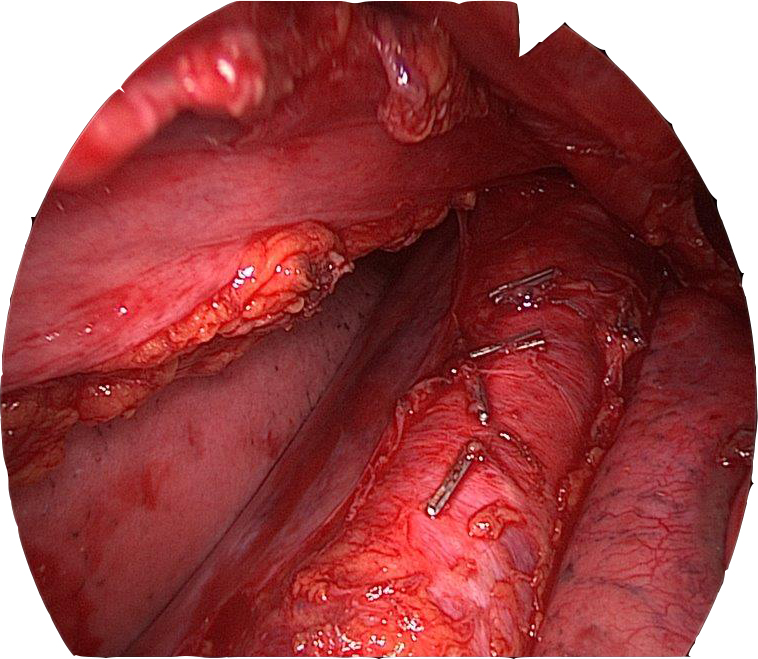


*aorta; **pericardium; ***left lung

Supplement: Supplementary file 2 — Additional file 2: Supp. Fig. S2. En bloc resection of the esophagus and periesophageal tissue, including all tissue between the aorta and pericardium (and the pleura, bilaterally). [file 12893_2020_855_MOESM2_ESM.docx]

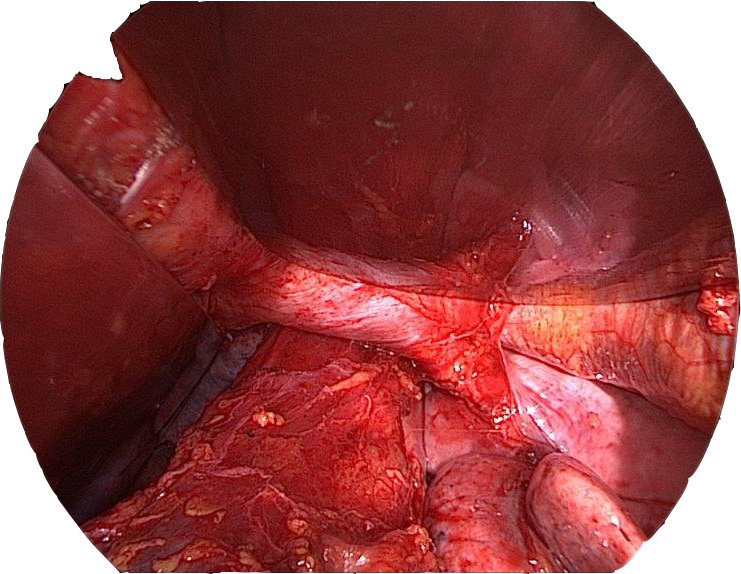


***

**

*

*lung hook; **esophagus;***left lung

Supplement: Supplementary file 3 — Additional file 3: Supp. Fig. S3. View of pulmonal arteries completely cleared from periesophageal tissue by elevating the pulmonary hilus anteriorly to gain access to the hilar structures, esophagus, and left lung. [file 12893_2020_855_MOESM3_ESM.docx]

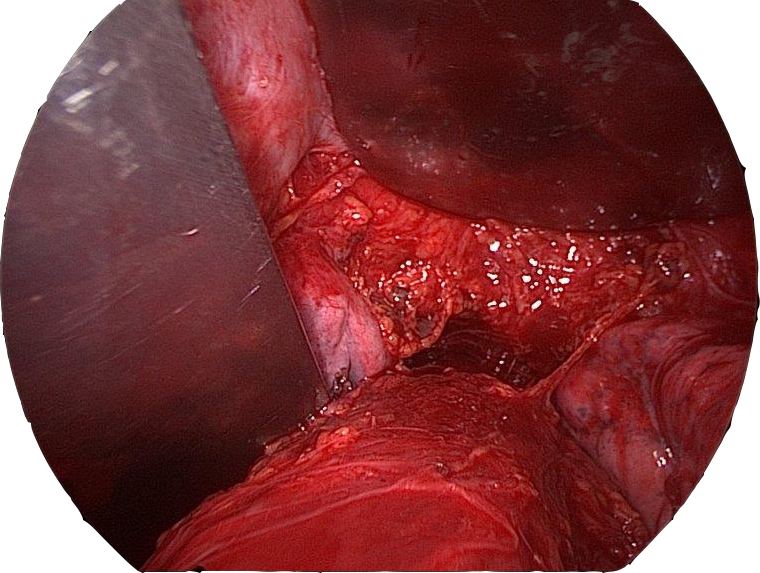


*

*esophagus

Supplement: Supplementary file 4 — Additional file 4: Supp. Fig. S4. View of the tracheal bifurcation/aortopulmonal window. [file 12893_2020_855_MOESM4_ESM.docx]
